# Using an UPLC/MS-based untargeted metabolomics approach for assessing the antioxidant capacity and anti-aging potential of selected herbs†

**DOI:** 10.1039/d0ra06047j

**Published:** 2020-08-26

**Authors:** Mohamed A. Salem, Rasha Ali Radwan, Eman Sherien Mostafa, Saleh Alseekh, Alisdair R. Fernie, Shahira M. Ezzat

**Affiliations:** Department of Pharmacognosy, Faculty of Pharmacy, Menoufia University Gamal Abd El Nasr St. Shibin Elkom 32511 Menoufia Egypt mohamed.salem@phrm.menofia.edu.eg; Biochemistry Department, Faculty of Pharmacy, Sinai University East Kantara Branch New City El Ismailia 41611 Egypt; Department of Pharmacognosy, Faculty of Pharmacy, October University for Modern Sciences and Arts (MSA) Giza 12451 Egypt shahira.ezzat@pharma.cu.edu.eg +20-120-000-4301; Max Planck Institute of Molecular Plant Physiology Am Mühlenberg 1 14476 Potsdam-Golm Germany Alseekh@mpimp-golm.mpg.de Fernie@mpimp-golm.mpg.de; Center for Plant Systems Biology and Biotechnology 4000 Plovdiv Bulgaria; Department of Pharmacognosy, Faculty of Pharmacy, Cairo University Cairo 11562 Egypt

## Abstract

Aging is an unavoidable fate that afflicts all life, during this process in mammals reactive oxygen species (ROS) are generated which stimulate tyrosinase, elastase and collagenase activities that actively participate in skin aging. Therefore, the maintenance of antioxidant homeostasis is an important anti-aging strategy for skin. Nature has excellent anti-aging remedies that act externally as well as internally to delay the visual signs of aging. In view of this fact, the present study investigates the *in vitro* anti-aging activity of five medicinal plants belonging to phenolic rich families namely *Rosmarinus officinalis*, *Lavandula officinalis*, *Matricaria chamomilla*, *Camellia sinensis* and *Pelargonium graveolens*. The selected plants are those most frequently used in the preparation of ethnomedicinal recipes for the prevention or treatment of aging. The inhibitory effects of the ethanolic and aqueous extracts of the five selected plants on the activity of tyrosinase, elastase, and collagenase enzymes were investigated. Furthermore, the chemical composition of the plants and the antioxidant capacity of their extracts were assessed. The results showed that *R. officinalis* had the highest total phenolics content which was correlated with its potent antioxidant and anti-aging activities. To pinpoint the active metabolites in the tested extracts, we evaluated the metabolite variations using ultra-performance liquid chromatography coupled with high resolution electrospray ionization-tandem mass spectrometry (UPLC-HR-ESI-MS/MS). Multivariate data analysis (MVDA) revealed that *R. officinalis* significantly accumulated metabolites from the aromatic diterpenoid, flavonoid and phenolic acid classes. These results indicate that rosemary can be used for further development of topical preparations with anti-aging properties.

## Abstract 2

Aging is an unavoidable fate that afflicts all life, during this process in mammals, reactive oxygen species (ROS) are generated which stimulate tyrosinase, elastase and collagenase activities that actively participate in skin aging.

## Graphical abstract

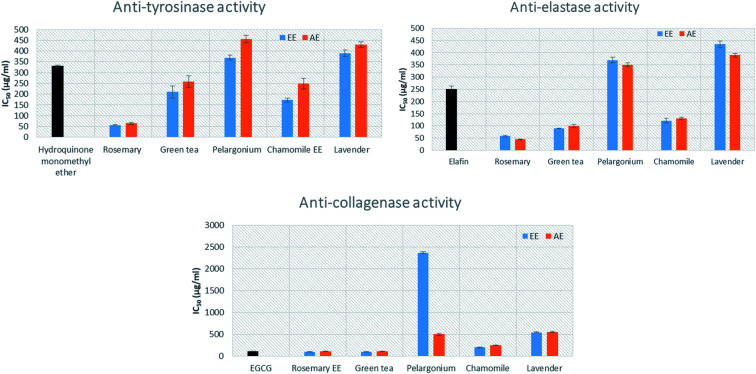

## 1. Introduction

Aging is an unavoidable process that all organisms must face. The effect of aging on the human body appears very clearly on the skin. Aging can be categorized into two main types: age-dependent/chronological aging (intrinsic aging) and premature aging/photoaging which is also known as extrinsic aging.^1^ Intrinsic aging appears by the effect of time, due to genetic factors and most commonly due to the imbalance between the natural antioxidants and the generated free radicals, all these factors may contribute to the appearance of aging alongside hormonal changes that can also cause wrinkling of the skin.^2^ On the other hand, sunlight, especially ultraviolet (UV) radiation plays a crucial role in photoaging or extrinsic skin aging.^3^ Photoaging usually causes a leathery appearance of the skin, dark/light pigmentation and deep furrows.^4^

The human body is continuously exposed to oxidative stress (OS) resulting from the excessive reactive oxygen species (ROS) such as superoxide anions, hydroxyl radicals and hydrogen peroxide on one side and disturbed antioxidant protection machinery on the other side.^5,6^ Excessive OS and ROS generation contributes to DNA mutation, protein damage, cellular apoptosis, inflammation and tissue damage leading to the progression of aging.^6^ OS and ROS play major roles in the aging of skin.^7^ Skin aging occurs as a result of the damage of its outermost layer, known as the extracellular matrix (ECM), this layer contains collagen and elastin which are crucial for skin integrity and elasticity.^8,9^ Activation of some enzymes such as collagenase, hyaluronidase and elastase are responsible for damage of ECM which directly causes the appearance of skin aging.^8,10^ Skin strength and elasticity depend mainly on collagen that is considered as the skin building unit. Activation of such enzymes, cause a decrease in the collagen and elastin levels in the skin with a resultant loss of its strength and elasticity and the development of aging.^1^

Daily exposure to sunlight especially in middle-aged individuals, can also cause increase in the production of melanin pigment which may lead to skin darkening and hyperpigmentation. This process may be stopped through the inhibition of tyrosinase enzyme, the key enzyme in synthesis of melanin.^11^ When the skin absorbs UV radiation, ROS generation increased with induction of oxidative stress, which leads to the activation of tyrosinase, elastase and collagenase enzymes with further appearance of skin aging.^1,12,13^

Herbs are invaluable sources of secondary metabolites that can be incorporated in sunscreens and skin lightener preparations. Many *in vitro* experiments have proven the efficacy of many herbs as elastase, tyrosinase and collagenase inhibitors.^14–16^

African people, in particular, suffer from harsh sun, and for these peoples skin care preparations are too expensive. Accordingly, the authors have seen that exploring plants for drug discovery of anti-aging preparations is essential. Five plants, that are most frequently used in the preparation of ethnomedicinal recipes, were chosen to evaluate their anti-collagenase, anti-tyrosinase, anti-elastase and anti-oxidant activities. The selection of the five plants was dependent on the reported use against wound healing, skin ageing or due to their antioxidant activity. Lavender (*Lavandula officinalis* L., f. Lamiaceae) and chamomile (*Matricaria chamomilla* L., f. Asteraceae) are used to enhance wound healing.^17,18^ Pelargonium (*Pelargonium graveolens* L., f. Geraniaceae) was reported to have antioxidant activity.^19^ Green tea leaves (*Camellia sinensis* L., f. Theaceae) contain catechins which possess a protective effect against skin ageing.^20^ In addition, rosemary (*Rosmarinus officinalis* L., f. Lamiaceae) was reported to have anti-aging activity.^21^ However, there is lack of information in the context of the application of these herbs as anti-aging agents. The chemical profile of these plants have not been compared and correlated to the anti-aging potential.

The application of sophisticated modern analytical techniques for profiling of metabolites in different biological systems has gained growing interest in the last decades.^22^ Metabolomics has emerged as a versatile tool for comprehensive analysis of the metabolome for food, nutraceutical, medicinal and pharmaceutical applications.^23^ Metabolomics studies can be performed using either targeted or untargeted approaches.^24^ In targeted metabolomics approaches, a specific set of known metabolites is analyzed in a quantitative or semi-quantitative manner.^25^ By contrast, untargeted metabolomics achieves the comprehensive analysis of all measurable analytes without necessarily identifying all compounds.^26^ Due to its comprehensive nature, datasets generated from untargeted metabolomics are subjected to chemometric methods, such as multivariate data analysis (MVDA), to reduce data complexity.^27^ MVDA achieves pattern-recognition through application of supervised or unsupervised methods.^28^ In unsupervised analysis methods such as principal component analysis (PCA), hierarchical clustering analysis (HCA) and self-organizing maps (SOMs), the similar patterns are identified without considering the labels of the study samples.^29^ By contrast, the supervised methods such as partial least squares (PLS) use the sample labels to assign feature(s) to a phenotype of interest.^30^

Accordingly, in this work, the aqueous and ethanolic extracts of *R. officinalis*, *L. officinalis*, *M. chamomilla*, *C. sinensis* and *P. graveolens* were tested for their inhibitory effect on tyrosinase, elastase and collagenase enzymes in correlation with their antioxidant potentiality. Ethanolic and aqueous extracts of *R. officinalis* exhibited the highest phenolic content and antioxidant activity that were positively correlated with the highest anti-aging activity. Moreover, an untargeted metabolomics approach was adopted to investigate and compare the chemical profiles of the tested extracts. This comprehensive metabolite analysis was achieved using ultra performance liquid chromatography coupled with high resolution electrospray ionization-tandem mass spectrometry (UPLC-HR-ESI-MS/MS). The subsequent chemical profiling and chemometric analysis have led to the identification of a range of metabolites, mostly represented by diterpenoids, coumarins and flavonoids. The identified metabolites were detected at higher levels in *R. officinalis* and were correlated to the antioxidant capacity and anti-aging potential.

## 2. Materials and methods

### 2.1 Chemicals and reagents

Gallic acid and folin ciocalteu as well as chemicals for ORAC assay such as sodium fluorescein, 2,2′-azobis(2-amidinopropane)dihydrochloride (AAPH), 6-hydroxy-2,5,7,8-tetramethylchroman-2-carboxylic acid (Trolox®), Nunc Micro-well™ plates, phosphate buffer (pH 7.4) were purchased from Sigma Aldrich. ORAC experiment was performed on fluorometer, FLUOstar OPTIMA, Franka Ganske, BMG LABTECH, Offenburg, Germany. DPPH free radical solution, vitamin C (ascorbic acid), mushroom tyrosinase enzyme obtained from mushroom, l-3,4-dihydroxyphenylalanine (l-DOPA), potassium dihydrogen orthophosphate (pH 6.5), potassium hydroxide, hydroquinone monomethyl ether, elastase enzyme, substrate: (*N*-methoxy-succinyl-Ala-Ala-Pro-Val-*P*-nitro-anilide), tris(hydroxymethyl)-methyl-2-aminoethane sulfonate (TES) buffer, HEPES buffer which is abbreviation for (4-2-hydroxyethyl-l-piperazine ethane sulfonic acid), pH = 7.5, elafin positive control, collagenase type 1 from *Clostridium histolyticum*, FALGPA substrate (*N*-[3-(2-furyl)acryloyl]-Leu-Gly-Pro-Ala) and epigallocatechin gallate (EGCG) were purchased from Sigma Aldrich. UV recordings were made on ELX 808 (Bio Tek Instrumental, Italy), plates were purchased from (Mekkawy, Egypt).

### 2.2 Plant material

The aerial parts of rosemary (*Rosmarinus officinalis* L.), lavender (*Lavandula officinalis* L.), chamomile (*Matricaria chamomilla* L.), pelargonium (*Pelargonium graveolens* L.) and the leaves of green tea (*Camellia sinensis* L.) were obtained from the Experimental Station of Medicinal and Aromatic Plants, Pharmacognosy Department, Faculty of Pharmacy, Cairo University, Giza, Egypt. The plants were kindly authenticated by Prof. Dr Wafaa Amer, Department of Botany, Faculty of Science, Cairo University, Giza, Egypt.

### 2.3 Preparation of extracts

For the aqueous extracts, the dried plant material of each plant (50 g) was macerated in 1.2 L distilled water followed by boiling for 2 h followed by filtration.^31^ The extracts were subjected to lyophilisation, and the lyophilized residue were weighed to yield 20, 22, 18, 19 and 18 g for rosemary, chamomile, pelargonium, lavender and green tea, respectively. For the ethanolic extracts, the dried powder of each plant (50 g) were macerated in 1.2 L of 95% ethanol and heated under reflux conditions for 2 hours at 70 °C. The extracts were then filtered. The final extracts were filtered and the solvents were evaporated using the rotary evaporator.^31^ The yield was 17, 20, 21, 22 and 23 g for rosemary, chamomile, pelargonium, lavender and green tea, respectively.

### 2.4 Quantitative estimation of the total phenolic content

The total phenolic contents were determined spectrophotometrically adopting the folin–ciocalteu colorimetric method.^32^ Gallic acid stock solution was first prepared (1 mg mL^−1^ in methanol) and serial dilutions were prepared (20–280 μg mL^−1^) to construct the standard calibration curve. Each extract or the standard (10 μL) was mixed with 2% Na_2_CO_3_ (10 μL), and of 50% folin ciocalteu reagent (10 μL). Absorbance of the mixture was measured at 760 nm against a blank (methanol instead of the test solution). The results were calculated as gallic acid equivalents (GAE). Three measurements were used for each concentration.

### 2.5 Quantitative estimation of the total flavonoid content

Definite amount of each extract (125 μL) was mixed with 5% NaNO_2_ solution (75 μL) then, the mixture was left for 6 min. After adding aluminium trichloride (10%) (150 μL), the mixtures were incubated for 5 min, then 1 M NaOH (750 μL) was added. Using distilled water, the solution was adjusted to 2500 μL. The solution was incubated for 15 min then the absorbance was measured at 510 nm. The total flavonoids content was reported as mg catechin equivalent.^33^

### 2.6 DPPH radical scavenging assay

The estimation of radical scavenging activity was done according to the method of ref. 34. DPPH (2,2-diphenyl-l-picrylhydrazyl), a stable radical, is reduced after reaction with an antioxidant compound and its absorbance at 517 nm is then reduced. The reaction mixture contained 500 μL of each extract, 375 μL ethanol and 125 μL of a 1 mm freshly prepared DPPH solution in ethanol. Different concentrations of test samples were prepared while the final concentration of DPPH in the reaction mixture was 0.125 mM. The tested samples were incubated in the dark for 30 min at 37 °C. Blank samples contained the same amount of methanol and DPPH solution. All measurements were carried out in triplicate. Ascorbic acid was used as a reference standard. Percentage radical scavenging activity of samples was calculated using the radical scavenging activity:(%) = (*A*_blank_ − *A*_sample_/*A*_blank_) × 100

IC_50_ values were calculated from the graph plotted for the concentration in μg mL^−1^ against the percentage inhibition.

### 2.7 ORAC radical scavenging assay

Tested samples were mixed with phosphate buffered saline (10 mM, pH 7.4) and investigated for their antioxidant activity. The time course for the fluorescein decay induced by AAPH compared to the positive control Trolox was used to evaluate the antioxidant capacity.^35,36^

### 2.8 Determination of anti-tyrosinase activity

Inhibition of tyrosinase was determined using Rauniyar *et al.*^37^ method, hydroquinone monomethyl ether was used as a standard. Tyrosinase enzyme (5600 units per mL) (80 μL) was mixed with 80 μL of 1 mg mL^−1^ hydroquinone monomethyl ether or the extract solution in DMSO incubated at 37 °C for 15 min. Then l-3,4-dihydroxyphenylalanine (l-DOPA) (40 μL) was added and incubated at 37 °C for 30 min to obtain 1 mM of l-DOPA as the final concentration. Absorbance of dopachrome was read at 475 nm ELX 808 (Bio Tek Instrumental, Italy). Blanks without any inhibitor/samples served as control. Hydroquinone monomethyl ether was used as a standard. Percentage of inhibition is calculated using the formula: 100 − [(*A*_sample_/*A*_control_) × 100].

### 2.9 Determination of anti-elastase activity

The anti-elastase activity was assessed in accordance with the method of Kraunsoe *et al.*,^38^ with minor modifications. 25 μL of human leukocyte elastase (1 μg mL^−1^) was mixed with 25 μL of the extract or standard (1.4 mg mL^−1^ in DMSO) and 25 μL of HEPES buffer (pH 7.5). The samples were incubated at 25 °C for 20 min. After adding the substrate *N*-methoxysuccinyl-Ala-Ala-Pro-Val-*p*-nitroanilide (1 mM), the mixture was incubated for 40 min at room temperature. Absorbance was measured at 405 nm using ELX 808 (Bio Tek Instrumental, Italy). Elafin was used as standard using the following concentration range (1–10 μg mL^−1^). Blank samples without any inhibitor/samples served as control.

### 2.10 Determination of anti-collagenase activity

To determine the anti-collagenase activity, 25 μL of collagenase enzyme (1 mg mL^−1^), 25 μL of TES buffer (50 mM) and 0.36 mM calcium chloride (pH 7.4) were mixed with the extract or standard (1.4 mg mL^−1^ in DMSO) and incubated in at 37 °C for 20 min.^39^ After adding 100 μL of FALGPA, the mixtures were incubated for 60 min at 37 °C. Equal volumes of 200 mM citrate buffer (pH 5) and ninhydrin solution were mixed and 200 μL of this solution was added. Further incubation at 100 °C for 5 min was done, and then 200 μL of 50% isopropanol was added after cooling to left to room temperature. Epigallocatechin gallate (EGCG) was used as standard using the following concentration range (1–10 μg mL^−1^). Blank samples without any inhibitor/samples serves as control. Absorbance was detected at 540 nm using ELX 808 (Bio Tek Instrumental, Italy). The percentage inhibition was calculated as for elastase.

### 2.11 UPLC-MS/MS analysis

The metabolites of the extracts were analyzed using RP High Strength Silica (HSS) T3 C18 column (100 mm × 2.1 mm containing 1.7 μm diameter particles, Waters), using a Waters Acquity UPLC system.^40^ The mass spectra were acquired by full scan MS in both postive and negative ionization modes on an exactive high resolution orbitrap-type MS (Thermo Fisher, Bremen, Germany).^41^

### 2.12 Statistical analysis

All the measurements were done in triplicate to obtain the mean values and standard deviations (SDs). The IC_50_ values were calculated from the linear regression curve constructed of the percentage inhibition on the *y*-axis and extracts concentrations on the *x*-axis.^42^ All analysis were done using the SPSS v. 22.0 (IBM, Chicago, USA). Microsoft Excel 2010 was applied for graph construction. All the obtained data from metabolite profiling were analyzed and correlated using metaboanalyst (https://www.metaboanalyst.ca/).^43^

## 3. Results

### 3.1 Determination of the anti-aging activity of the tested extracts

**Fig. 1:**
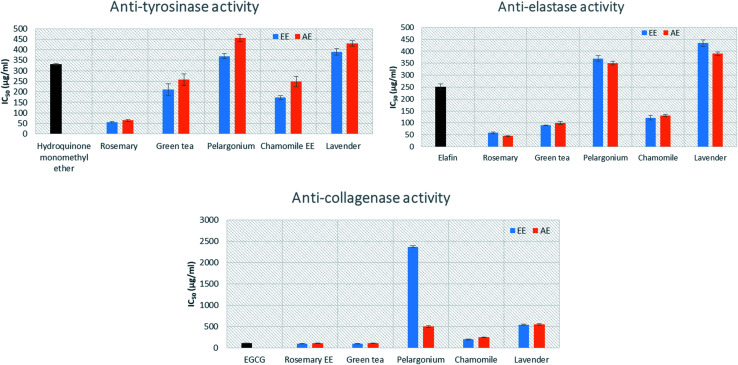
Anti-tyrosinase, anti-elastase and anti-collagenase activities of rosemary, lavender, green tea, pelargonium and chamomile aqueous (AE) and ethanoic (EE) extracts. The results are expressed as the mean ± SD, *n* = 3.

The anti-tyrosinase activity of the aqueous (AE) as well as the 95% ethanoic (EE) extracts of the selected herbs (lavender, chamomile, pelargonium, green tea and rosemary) was evaluated based on the conversion of l-DOPA to dopaquinone by mushroom tyrosinase.^44^ The results indicated that rosemary AE and EE showed the highest inhibitory activity of tyrosinase (IC_50_ = 65.03 ± 4.88 and 55.03 ± 3.88 μg mL^−1^, respectively), compared to the standard hydroquinone monomethyl ether (with IC_50_ = 330.0 ± 4.1) (Fig. 1). The EE of chamomile, AE and EE of green tea (IC_50_ = 172.44 ± 10.1, 257.18 ± 27.19 and 210.25 ± 28.1 μg mL^−1^, respectively), showed lower inhibitory activity than rosemary but stronger activity than that of the standard. Pelargonium was shown to exhibit the lowest inhibitory activity (IC_50_ = 455.27 ± 18.15 μg mL^−1^).

The effects of the extracts on elastase activity indicated that the AE and EE of rosemary showed the highest inhibition activity (IC_50_ = 45.11 ± 2.11 and 58.22 ± 3.21 μg mL^−1^, respectively), followed by the AE and EE of green tea (IC_50_ 100 ± 6.43 and 90 ± 1.34 μg mL^−1^) (Fig. 1). Moreover, the AE and EE of chamomile displayed significant activity with IC_50_ = 130.29 ± 5.09 and 121.55 ± 10.1 μg mL^−1^, respectively, when compared to the standard elafin (IC_50_ = 250.0 ± 12.6 μg mL^−1^). Additionally, the EE of rosemary showed the strongest inhibition activity of collagenase enzyme with IC_50_ = 100.32 ± 3.33 μg mL^−1^, followed by EE of green tea (IC_50_ 100.1 ± 3.89 μg mL^−1^). This activity was comparable to the standard epigallocatechin gallate (EGCG) (IC_50_ = 112.12 ± 9.4 μg mL^−1^).

### 3.2 Determination of radical scavenging activity of the tested extracts

**Fig. 2:**
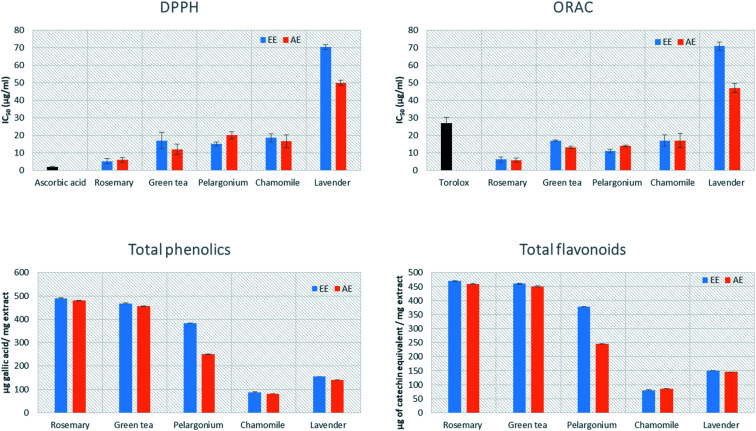
Antioxidant capacity, total phenolics, flavonoids content of rosemary, lavender, green tea, pelargonium and chamomile aqueous (AE) and ethanoic (EE) extracts. The results are expressed as the mean ± SD, *n* = 3.

The antioxidant activity of the tested extracts was measured by 2,2-diphenyl-1-picrylhydrazyl (DPPH) and oxygen radical absorbance capacity (ORAC) assays. DPPH, which is the most commonly used assay for evaluation of the antioxidant activity of plant extracts, is based on the discoloration produced when an antioxidant reacts with the stable free radical DPPH˙.^45^ All the tested extracts were evaluated and compared to ascorbic acid, which has strong antioxidant properties, as a reference standard.^46^ The AE and EE of rosemary (IC_50_ = 5.90 ± 1.33 and 5.21 ± 1.37 μg mL^−1^, respectively) showed the highest activity, followed by the AE of green tea (IC_50_ = 12 ± 2.91 μg mL^−1^) (Fig. 2). The AE and EE of lavender showed the lowest antioxidant activity (IC_50_ = 50 ± 1.45 and 70.45 ± 1.32 μg mL^−1^, respectively).

The oxygen radical absorbance capacity (ORAC) assay measures the antioxidant capacity of a molecule based on the ability of AAPH (2,2′-azobis(2-methylpropionamidine)dihydrochloride) to generate peroxyl free radicals as ROS. A fluorescent signal from a probe is quenched in presence of ROS and persist in presence of antioxidants.^47^ All the tested extracts were evaluated and compared to Trolox, which has strong antioxidant properties, as a reference standard.^48^ The highest antioxidant activity was noted for the AE and EE of rosemary (5.75 ± 1.23 and 6.21 ± 1.48 μg mL^−1^, respectively), while the AE and EE of lavender showed the lowest antioxidant activity (47 ± 2.66 and 71 ± 2.33 μg mL^−1^) (Fig. 2).

### 3.3 Determination of total phenolics and flavonoids content

The total phenolic and flavonoid contents of the AE and EE of the five plants were determined spectrophotometrically using folin ciocalteu reagent and aluminium trichloride assay, respectively. The highest phenolic content was detected in both AE and EE of rosemary (480 ± 0.44 and 490 ± 0.55 μg of GAE per mg extract), followed by AE and EE of green tea (455.76 ± 0.49 and 467.3 ± 0.75 μg of gallic acid equivalent per mg extract) (Fig. 2). The highest flavonoid content was detected in the AE and EE extracts of rosemary (219.88 ± 0.01 and 260.8 ± 2.1 μg catechin equivalent per mg extract) (Table 1), followed by aqueous and ethanolic green tea extracts (450.76 ± 0.49 and 460.3 ± 0.75 μg catechin equivalent per mg extract). Intriguingly, the content of total phenolics was positively and significantly correlated with antioxidant capacity and *in vitro* anti-aging activity (Table 1).

**Table 1:** Pearson's correlation coefficients indicating the relationship between phenolics and *in vitro* anti-aging activity^a^

	Total phenolics	Total flavonoids	IC_50_ DPPH	IC_50_ ORAC	IC_50_ anti-tyrosinase	IC_50_ anti-elastase	IC_50_ anti-collagenase
Total phenolics	1						
Total flavonoids	0.999^a^	1					
IC_50_ DPPH	−0.569^b^	−0.563^b^	1				
IC_50_ ORAC	−0.542^b^	−0.536^b^	0.99^a^	1			
IC_50_ anti-tyrosinase	−0.48^b^	−0.459^b^	0.638^a^	0.56^b^	1		
IC_50_ anti-elastase	−0.47^b^	−0.458^b^	0.758^a^	0.69^a^	0.904^a^	1	
IC_50_ anti-collagenase	−0.012	−0.001^b^	0.092	0.029	0.478^b^	0.599^a^	1

a a and b indicate that the correlation is significant at *p* < 0.001 and 0.01, respectively.

### 3.4 UPLC-HR-ESI-MS/MS analysis and metabolite identification

In order to elucidate which metabolites discriminated the rosemary extract from other extracts in terms of high antioxidant potentiality and anti-aging activity, a non-targeted metabolomics approach was applied. Metabolic profiling of the EE and AE of the five plants was studied using UPLC-HR-ESI-MS/MS in both negative and positive ionization modes (Fig. S1–S5†). Different classes of phenolic compounds were identified in the ethanolic and aqueous extracts of the studied plants *viz.* flavonoids, hydroxycinnamic acids and their glycosides, coumarins, diterpenoids and fatty acids (Table S1†). Metabolites were identified by comparing the MS and MS/MS data to common MS databases such as Human Metabolome Database (http://www.hmdb.ca/), MassBank (www.massbank.jp) and METLIN (http://metlin.scripps.edu). An accuracy error of 10 ppm was set in the MS search and the fragments were verified in MS/MS search. Moreover, the characteristic molecular ion and corresponding fragments were confirmed to exhibit a pattern of co-elution.

**Fig. 3:**
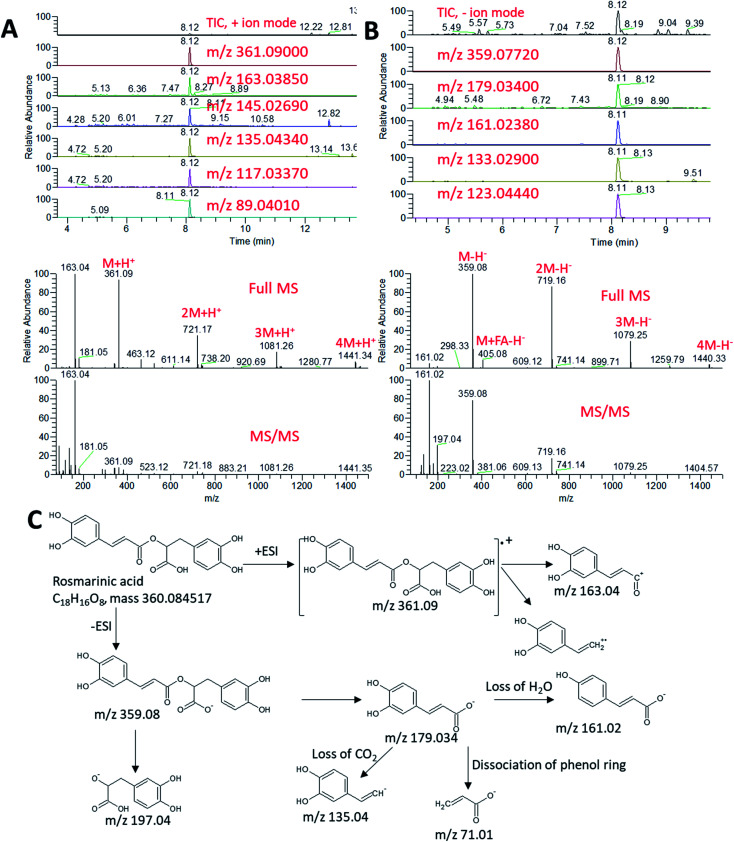
Total ion chromatogram (TIC) and extracted ion chromatograms (EIC) of the peak at *m*/*z* representing rosmarinic acid measured by UPLC/MS positive (A) and negative (B) ionization mode. Full MS and MS/MS spectra are shown. (C) Schematic diagram showing the production of fragment ions from rosmarinic acid during MS/MS analysis.

An exemplary description for the identification of rosmarinic acid is shown in Fig. 3. The extracted ion chromatograms (EIC) of its protonated adduct was detected at *m*/*z* 361.09 [M + H]^+^ and a retention time of 8.12 min. The predicted molecular formula for this adduct was C_18_H_17_O_8_. The MS spectra of the compound showed dimer, trimer and tetramer molecules that also co-eluted at the same retention time (Fig. 3A). In negative ionization mode, a deprotonated adduct at *m*/*z* 359.07 [M − H]^−^ eluted at the same retention time. The predicted molecular formula for this adduct was C_18_H_15_O_8_ (Fig. 3B). A deprotonation followed by addition of formic acid [M + FA − H]^−^ at *m*/*z* 405.08 was also detected together with dimer, trimer and tetramer molecules. Searching the molecular formula against common MS databases showed that the formula could be tentatively assigned to rosmarinic acid. Using the information from tandem mass spectrometry, confirmation of the compound identification was based on the detection of the characteristic fragment ions. For instance, the loss of water molecule from the protonated adduct was detected at *m*/*z* 343.08 in positive ionization mode. A caffeic acid fragment was detected at *m*/*z* 179.03 in negative ionization mode together with its dissociation (Fig. 3C). The loss of water and carbon dioxide from the caffeic acid fragment gave characteristic ions at *m*/*z* 161.02 [M − H − H_2_O]^−^ and *m*/*z* 135.04 [M − H − COO]^−^, respectively.

Using the same approach, all other metabolites were identified by their accurate mass measurements and MS/MS spectral data (Table S1†). Among the identified compounds, GMCA [2-β-d-glucopyranosyloxy-4-methoxycinnamic acid] was a major phenolic detected in the negative mode at Rt 5.69 min with [M − H] at *m*/*z* 355.103. Tonghaosu and α-bisabolol oxide C were detected peaks in the positive mode with [M + H] at *m*/*z* 201.0905 and 239.2000, respectively. In the negative mode at Rt 8.42 min, a peak with [M − H] at *m*/*z* 193.0497 and [M + H] at *m*/*z* 195.06505 identified as ferulic acid, while 4-methylumbelliferone showed abundant peak detected in the positive mode at Rt 9.78 min at *m*/*z* 177.0545 for [M + H].

At Rt 7.12 min in the negative mode, a peak was detected showing [M − H] at *m*/*z* of 463.08807 and was also detected in the positive mode as [M + H] of 465.102 and identified as quercetin 3-*O*-glucoside, while kaempferol 3-d-galactoside was detected at Rt 7.97 min with [M − H] at 447.09375. *O*-Coumaric acid 2-glucoside was detected at Rt 5.10 min with [M − H] at *m*/*z* 325.0925 and [M + H] at *m*/*z* of 327.10672. Some volatile constituents such as carylophyllene oxide was detected at Rt 14.31 with [M + H] at *m*/*z* 207.17412. Tannin derivatives such as glucogallic acid, theogallin, epigallocatechin, epigallocatechin gallate and catechin gallate were identified and were characteristic to green tea. The positive mode analysis of both the aqueous and ethanolic extracts of green tea showed one major peak at Rt 5.27 with [M + H] at *m*/*z* of 195.0875 which was identified as caffeine. An abundant peak at Rt 13.89 with [M − H] at *m*/*z* 329.1753 and [M + H] at *m*/*z* 331.1896 was identified as the phenolic diterpene carnosol. Another peak at Rt 14.54 min with [M − H] at *m*/*z* 331.1911 and [M + H] at *m*/*z* 333.2054 identified as the benzenediol abietane diterpene carnosic acid. A major peak at Rt 8.12 min with [M − H] at *m*/*z* 359.0728 was identified as rosmarinic acid.

### 3.5 Classification of the tested extracts by multivariate data analysis

**Fig. 4:**
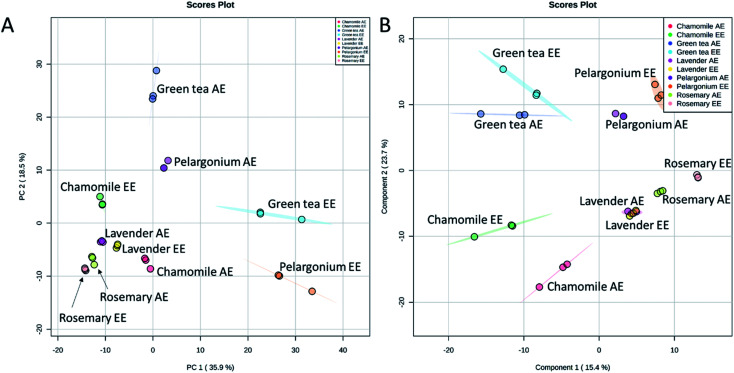
Score plots of PCA (A) and PLS-DA (B) based on the UPLC/MS data from rosemary, lavender, green tea, pelargonium and chamomile aqueous (AE) and ethanoic (EE) extracts.

The chemical profiles of the analyzed extracts from the selected plants were compared by the non-targeted UPLC/MS analysis coupled to chemometrics analysis. Principal component analysis (PCA) is a non-supervised multi-variate statistical method used to identify the directions of maximum data variability. PCA analysis of the extracts from the five selected plants revealed that the first two PCs explained 54.4% of the total variance (Fig. 4A). Lavender, rosemary and chamomile extracts were clustered from pelargonium which was also clustered from green tea. Along the PC2, the aqueous and ethanoic extracts of pelargonium and green tea were discriminated (Fig. 4A). In contract to PCA, partial least squares-discriminant analysis (PLS-DA) is a supervised method that takes into account the class labels for achieving dimensionality reduction. When PLS-DA was applied, a significant grouping of the tested extracts as a function of their species was obtained (Fig. 4B). Aqueous and ethanolic extracts of lavender were undistinguishable from one another and were closest to aqueous and ethanolic extracts of rosemary (Fig. 4B). Aqueous and ethanoic extracts of chamomile were very distinct from one another.

**Fig. 5:**
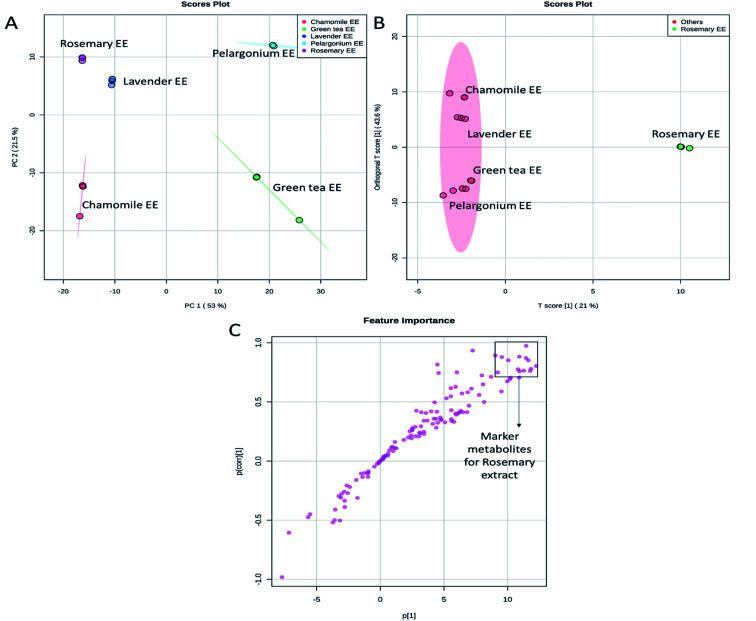
Score plots of PCA (A), OPLS-DA (B) and *S*-plot of OPLS-DA (C) based on the UPLC/MS data from rosemary, lavender, green tea, pelargonium and chamomile ethanoic (EE) extracts.

Since the aqueous and ethanolic extracts did not show significant differences in the extracts having high phenolic contents, antioxidant capacity and skin ageing inhibiting characters (Fig. 1 and 2), we focused our further analysis on the ethanolic extracts. PCA analysis of the ethanolic extracts from the five selected plants revealed that the first two PCs explained 74.5% of the total variance (Fig. 5A). Lavender, rosemary and chamomile extracts were discriminated along the PC1 from pelargonium and green tea. PC2 revealed discrimination of all selected extracts. Lavender and rosemary were closest to one another.

To determine the metabolites discriminating rosemary extract, which showed the highest phenolic contents, antioxidant potential and anti-aging activity, from other extracts, a comparative analysis was performed using the supervised Orthogonal Projections to Latent Structures Discriminant Analysis (OPLS-DA) (Fig. 5B). The OPLS-DA loadings *S*-plot displayed the distinctive metabolites contributed to the discrimination of rosemary extract from other extracts. The marker metabolites accumulated in rosemary were directed to the positive side of the plot. In contrast, metabolites which discriminated other tested extracts were directed to the negative side (Fig. 5B). The distinctive metabolites that showed significant accumulation in rosemary with *p* values equal to or less than 0.01 were filtered and considered as distinctive biomarkers (Fig. 5C and Table S2†).

### 3.6 Correlation between metabolite content and skin-anti-aging activity

The metabolites responsible for discrimination of rosemary from the tested extracts were next investigated. Pearson's correlation coefficients indicating the relationships between metabolites and antioxidant capacity as well as anti-aging activity are summarized in Table 2. These metabolites represented three major classes of phenolic compounds; the diterpenoids abietatrienes, phenolic acids and glycosides of quercetin and luteolin.

**Table 2:** Pearson's correlation coefficients indicating the relationships between metabolites and antioxidant capacity as well as anti-aging activity^a^

Correlation	Anti-aging activity			Antioxidant capacity	
	Anti-tyrosinase	Anti-collagenase	Anti-elastase	DPPH	ORAC
11,12-Dimethylrosmanol	0.934^a^	0.622^c^	0.776^a^	0.769^a^	0.65^b^
6,7-Dimethoxy-7-epirosmanol	0.608^c^	0.361	0.311	0.227	0.104
7-Methylrosmanol	0.572^c^	0.169	0.227	0.286	0.216
Quercetin 3,3′-dimethyl ether 7-rutinoside	0.756^b^	0.416	0.458	0.394	0.296
Carnosic acid	0.873^a^	0.525^c^	0.603^c^	0.543^c^	0.43
Carnosol	0.598^c^	0.171	0.201	0.270	0.183
Luteolin *p*-coumarylglucoside	0.600^c^	−0.035	0.197	0.508	0.487
Diosmetin	0.661^b^	0.496	0.396	0.212	0.066
Epirosmanol	0.666^b^	0.399	0.430	0.349	0.257
Isorosmanol	0.381	−0.034	−0.030	0.120	0.048
Luteolin 3′-*O*-glucuronide	0.943^a^	0.816^a^	0.830^a^	0.591^c^	0.438
Luteolin acetylglucuronide	0.941^a^	0.824^a^	0.831^a^	0.573^c^	0.421
*p*-Coumaroylquinic acid	0.758^b^	0.430	0.476	0.401	0.306
Rosmaridiphenol	0.873^a^	0.341	0.572^c^	0.734^b^	0.663^b^
Rosmadial	0.467	0.016	0.075	0.205	0.157
Rosmanol	0.761^a^	0.490	0.459	0.362	0.218
Rosmarinic acid	0.448	0.297	0.150	0.059	−0.087

a a, b and c correlation is significant at the 0.001, 0.01 and 0.05 levels, respectively.

**Fig. 6:**
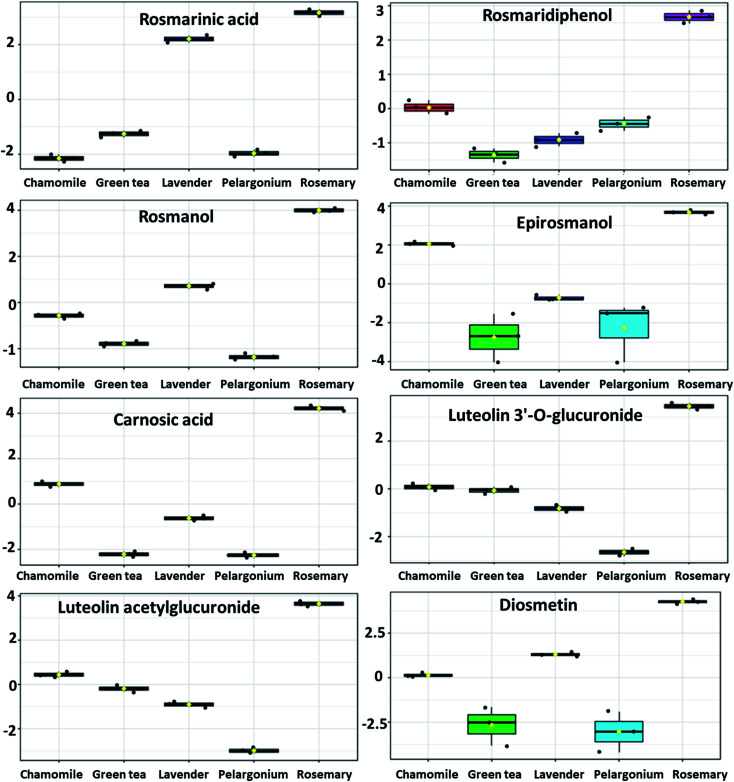
Metabolites with significant accumulation in rosemary ethanolic extract. The *y*-axis represents the scaled and log_2_-transformed values of metabolite abundance.

**Fig. 7:**
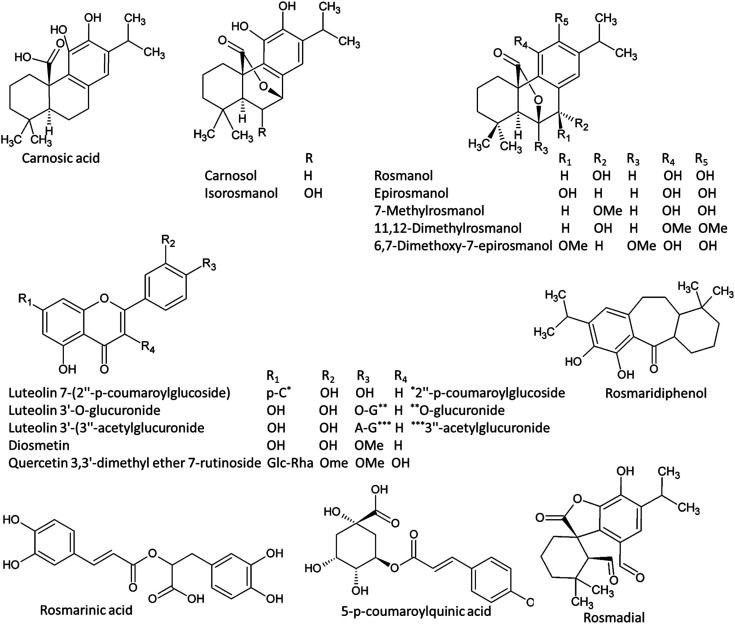
Chemical structured of metabolites with significant accumulation in rosemary ethanolic extract.

Among the phenolic abietatrienes, carnosic acid and its oxidation product carnosol showed significantly higher level in rosemary (Fig. 6 and 7). The principal degradation products of carnosic acid, rosmanol and its derivatives such as epirosmanol, isorosmanol, 7-methylrosmanol, 11,12-dimethylrosmanol, 6,7-dimethoxy-7-epirosmanol also showed relatively higher level in rosemary. The content of carnosic acid and its metabolites as well as the glycosides derivatives of the flavone luteolin were positively and significantly correlated with both antioxidant and *in vitro* skin anti-aging potentiality.

Cluster analysis based on the selected discriminating metabolites from the analyzed extracts demonstrated clear differences (Fig. 5D). Hierarchical cluster analysis (HCA) revealed two main clusters. The first cluster consisted of rosemary that was discriminated from other plant extracts. A clear diversification of lavender and chamomile from pelargonium and green tea could be observed in the second cluster.

## 4. Discussion

The development of cosmetic products for improving skin appearance or delaying skin aging has increased much attention in the recent years. Several researches have been directed to evaluate different plants for their skin anti-aging potentiality. Plant-derived antioxidant compounds can combat skin aging by reducing the abundance of ROS.^49^ Plant metabolites such as vitamins and phenolics, when applied topically, strengthen the skins protection system and reduce the imbalance between antioxidant machinery and ROS production.^50^ No reports were found dealing with the anti-aging activity of the five selected plants except for rosemary and green tea.^20,21^ Moreover, no detailed study was found to discuss their metabolite profile and its effect on their anti-aging activities.

In this study, a combination of *in vitro* anti-aging and antioxidant activities of plant extracts obtained from the alcohol and the aqueous extracts of five traditionally used plants was performed. Rosemary extracts showed promising results, when compared to the other species. Furthermore, the metabolite profiles of the tested extracts were also investigated to determine the correlation with the inhibitory potential on the tested enzymes. The concentration of total phenolics and particular phenolic classes correlated with the antioxidant capacity and the inhibition of skin aging related enzymes.

The antioxidant assays revealed that rosemary showed the highest DPPH and ORAC radical scavenging activities. *In vitro* antioxidant capacity can be evaluated by hydrogen atom transfer (HAT)- and electron atom transfer (ET)-based assays.^51^ DPPH and ORAC belong to ET and HAT assays, respectively.^52^ Therefore, DPPH assay is applied to quantify antioxidant's reducing capacity, while ORAC is applied to quantify peroxyl radical scavenging capacity.^53^ It can be anticipated that rosemary extracts have wide range of scavenging reactive oxygen species including peroxyl radicals. Increased ROS lead to activation of collagenase and elastase enzymes, which can further contribute to skin aging,^54^ therefore, the antioxidant activity of rosemary will increase its skin anti-aging activity.

The discriminating biomarkers from rosemary extracts contributed to the high total phenolic contents as well as showing positive correlation with the antioxidant activity and anti-aging potentiality. These metabolites represented three major classes of phenolic compounds; the diterpenoid abietatrienes, phenolic acids and the glycosides of quercetin and luteolin. Carnosic acid, a phenolic tricyclic abietatriene, has been detected as a major metabolite discriminating rosemary, showing also significant and positive correlation with anti-aging activity. Abietanes belong to the class of diterpenoids (C20) that have been isolated from a variety of natural sources, of which aromatic abietanes comprise the largest abietane group.^55^ Abietanes are components of extracts from many conifers as well as several angiosperms, particularly from the families Lamiaceae, Asteraceae, Celastraceae and Hydrocharitaceae.^55^ Abietanes play major roles in plants as defense metabolites; therefore, it is not surprising that they have been reported to exert anti-microbial, anti-leishmanial, anti-plasmodial, as well as antioxidant activities.^55,56^ Carnosic acid, a C-20 carboxylic acid, has been isolated from several Lamiaceae herbs including rosemary and sage (*Salvia officinalis*). Carnosic acid possesses very potent antioxidant activity owing to its *o*-diphenol structure. Next to its antioxidant activity, carnosic acid has been found to be effective for management of viral and bacterial infections, metabolic disorders, Alzheimer's disease and atherosclerosis. Carnosic acid inhibits collagen, arachidonic acid and thus exerts anti-inflammatory and anticancer activities.

From abietatrien 20-7 lactones, carnosol which is an oxidation product of carnosic acid as well as rosmanol and its derivatives such as epirosmanol, isorosmanol, 7-methylrosmanol, 11,12-dimethylrosmanol, 6,7-dimethoxy-7-epirosmanol accumulated significantly in rosemary. Abietatriene lactones, which have an additional oxygen-containing ring lactone (*i.e.* abietatrien-20,7-olides), have been isolated from several Lamiaceae herbs including rosemary and sage. These phenolic compounds have been reported to exert potential anti-microbial and anti-inflammatory activities.^55,57^ These diterpenoids have been shown to exhibit remarkably potent antioxidant activities compared to α-tocopherol.^57,58^

The underlying mechanisms of the *in vivo* and *in vitro* anti-inflammatory activity of carnosol and carnosic acid have been deeply investigated.^58,59^ Both compounds were shown to inhibit the formation of pro-inflammatory leukotrienes, reduce ROS formation and attenuate the secretion of human leukocyte elastase (HLE).^59^ The increase in HLE release is associated with damage in matrix proteins and thus increasing the risk of inflammation.^60^ Through reducing HLE secretions, carnosol and carnosic acid prevent the skin inflammations and attenuate aging.^59^ Leukotriene biosynthesis is initiated by the action of 5-lipoxygenase enzyme that transforms arachidonic acid to leukotriene A4.^61^ Excessive leukotrienes production is connected to numerous diseases including skin inflammation.^62^ Therefore, leukotriene blockage is considered a potential target for anti-inflammatory agents. The *O*-diphenolic diterpene carnosic acid, its oxidation product carnosol and other polyphenols encompassing flavonoids belong to the category of redox-active 5-lipoxygenase inhibitors.^63^ Since inflammatory responses promote age-related disorders, such natural compounds derived particularly from rosemary will likely attenuate skin aging.

Among other phenolic compounds, the diterpenoid rosmaridiphenol, the phenolic aldehyde rosmadial as well as the phenolic acids such as *p*-coumaroylquinic acid and the caffeic acid ester rosmarinic acid have been also accumulated in rosemary extract. The antioxidant activity of these compounds have been also reported.^64,65^ Flavonoids, particularly the glycosides of quercetin and luteolin which showed significantly higher level in rosemary comparted to other tested extracts, have been reported to attenuate skin aging by absorbing UVB, counteracting skin inflammation and/or inducing endogenous skin defense mechanisms.^66,67^ These flavonoids also exerted moderate antioxidant, anti-tyrosinase and anti-elastase activities suggesting their use as good candidates for skin aging.^68–72^

These results emphasize the synergistic and overall high effectiveness of these phenolic compounds and notably *R. officinalis* extract as anti-aging. By applying the provided metabolomics approach, rapid dereplication of previously known metabolites from the tested extracts was efficiently delivered. This was essential to avoid the redundant isolation of previously known compounds, and thus saving time, cost and protecting the environment from the non-green solvents. Nevertheless, biologically-guided fractionation of the total extracts will be essential for evaluating the effectiveness of these metabolites to attenuate skin aging *in vivo*, possibly by restoring the skin elasticity and thereby slow the wrinkling process.

## 5. Conclusion

Aging is a phenomenon that happens naturally and leads to different changes in the skin physiology. Nature has excellent anti-aging treatments that work externally and internally to delay signs of aging and some of which will work to prevent and repair signs of aging. In the present, the study of the five selected plants led to the selection of *R. officinalis* with powerful anti-oxidant activity against wrinkles and aging processes. In conclusion, we demonstrated that this anti-oxidant activity is due to their high phenolics and flavonoids contents. *R. officinalis* extract have the ability to inhibit elastase, collagenase, and tyrosinase enzymes. Furthermore, the LC-MS analysis has shown that the major compound of rosemary that showed significant accumulation and positive correlation with the biological activity were phenolics. In summary, our results reveal three groups among the main antioxidant compounds from *R. officinalis*; phenolic acids, flavonoids and diterpenoids, which have been reported before to have a high antioxidant activity. Given that rosemary is a cheap and non-toxic herb, the introduction of its phenolic-rich extracts into topical preparations for prevention or treatment of skin aging deserves further *in vivo* investigations.

## Conflicts of interest

No conflict to declare.

## Supplementary Material

RA-010-D0RA06047J-s001
