# Supplementary material for: Using an UPLC/MS-based untargeted metabolomics approach for assessing the antioxidant capacity and anti-aging potential of selected herbs
Source: RSC Adv. 2020 Aug 26;10(52):31511–24. doi: 10.1039/d0ra06047j (PMC9056420; doi:10.1039/d0ra06047j)
Supplement: RA-010-D0RA06047J-s001 [file RA-010-D0RA06047J-s001.pdf]

Electronic Supplementary Material (ESI) for RSC Advances.
This journal is © The Royal Society of Chemistry 2020

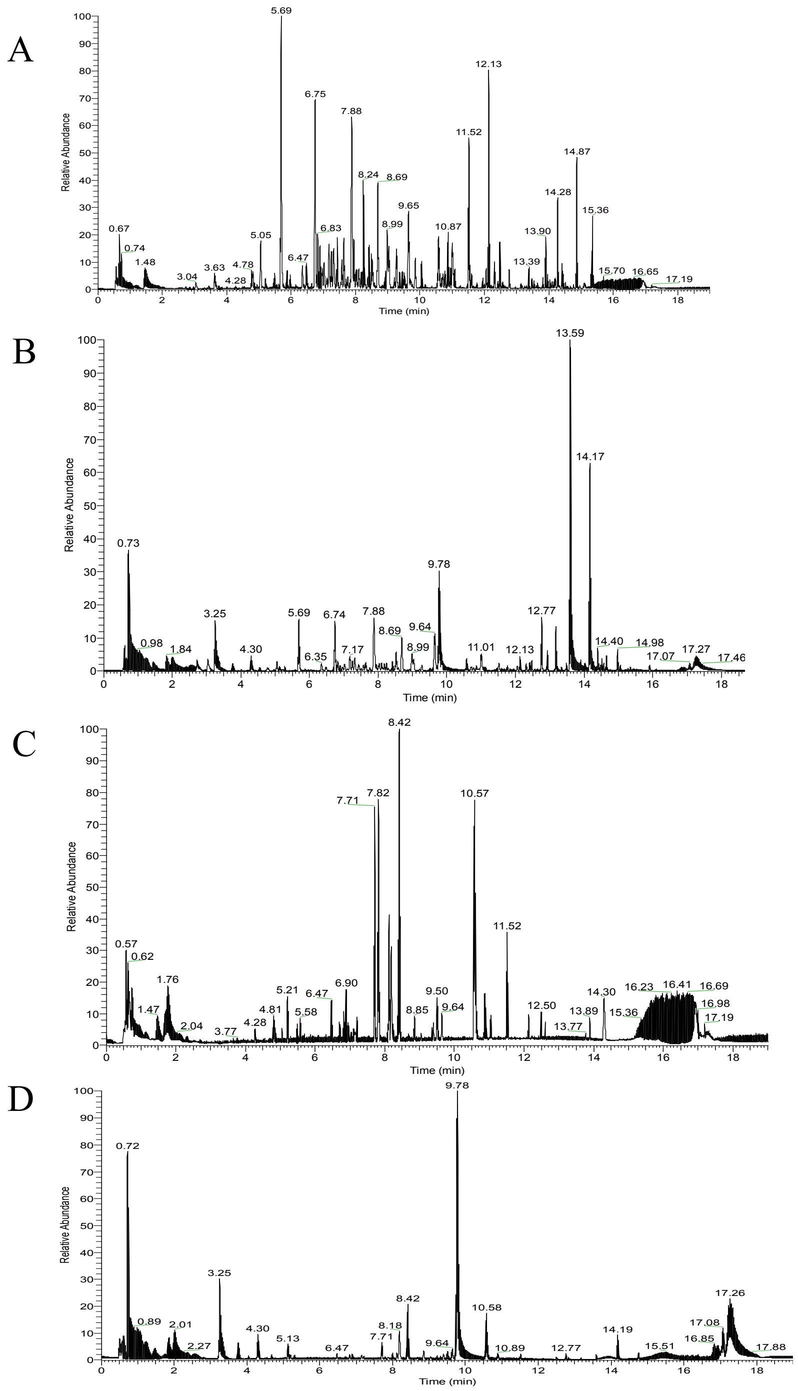

Figure S1. Base peak chromatogram of the ethanol extract of Chamomile, A: negative mode; B, positive mode and aqueous extract of chamomile, C, negative mode; D, positive mode.

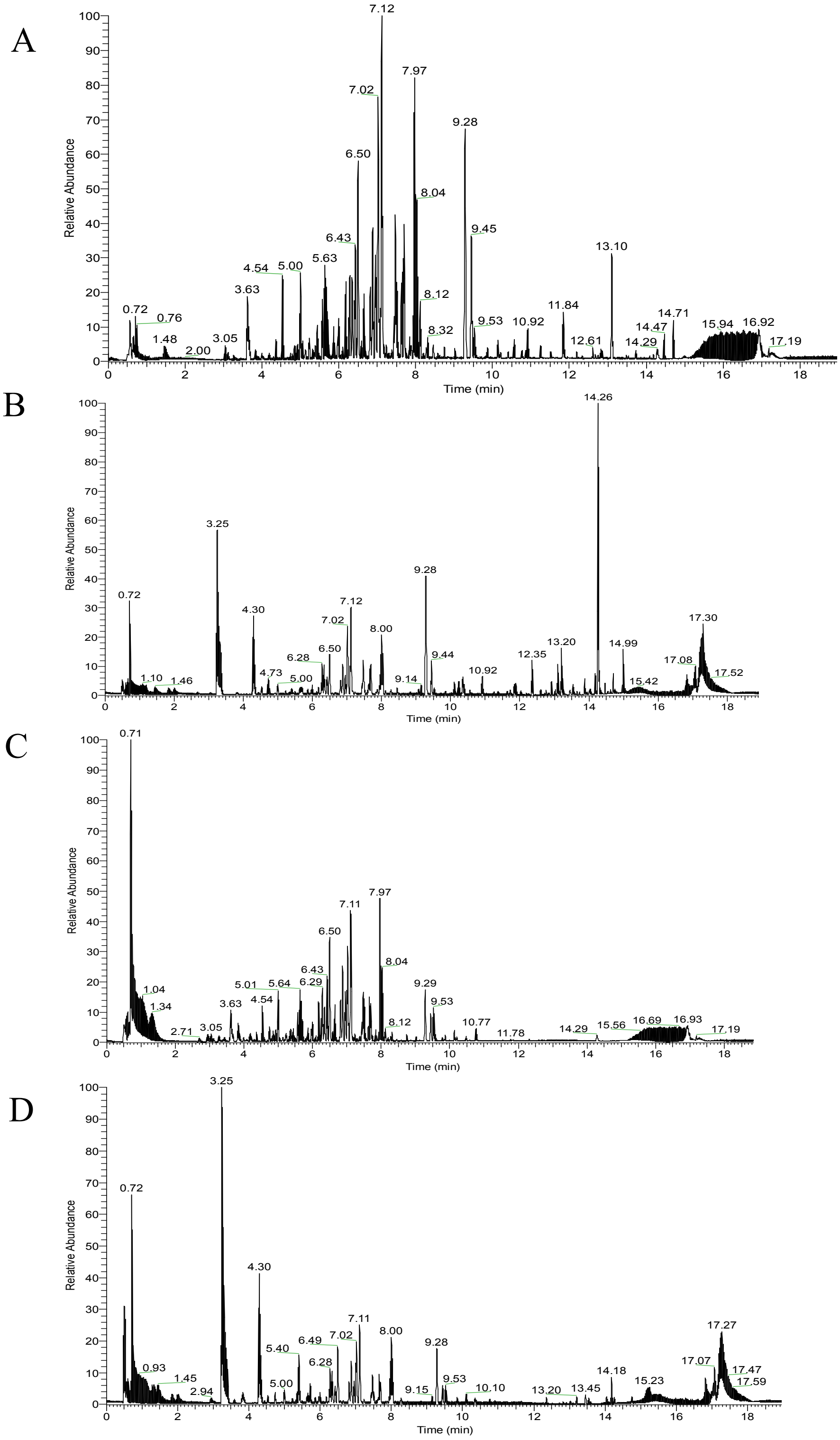

Figure S2. Base peak chromatogram of the ethanol extract of Pelargonium, A: negative mode; B, positive mode and aqueous extract of pelargonium, C, negative mode; D, positive mode.

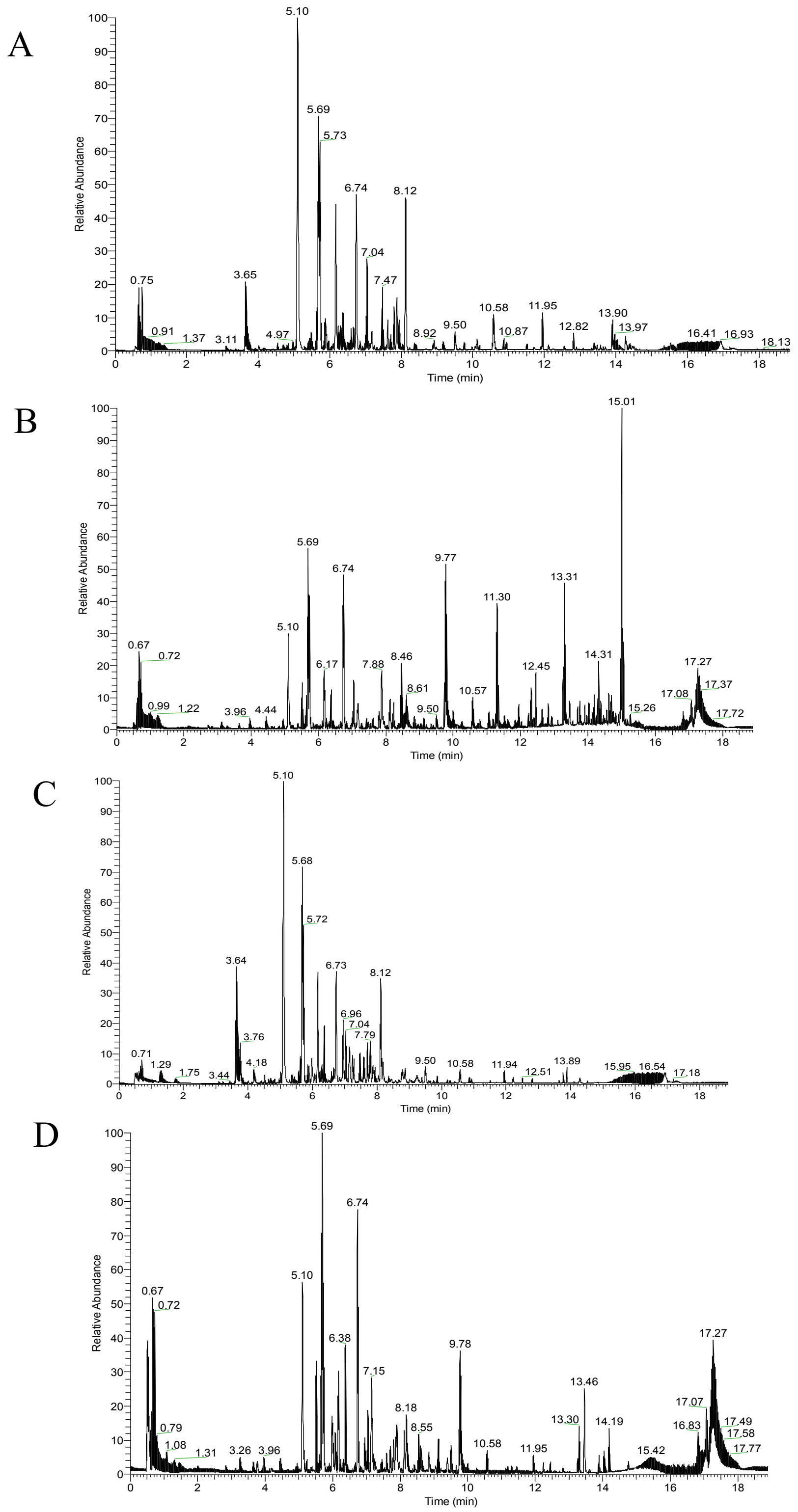

Figure S3. UPLC Base peak chromatogram of the ethanol extract of lavender, A: negative mode; B, positive mode and aqueous extract of lavender, C, negative mode; D, positive mode.

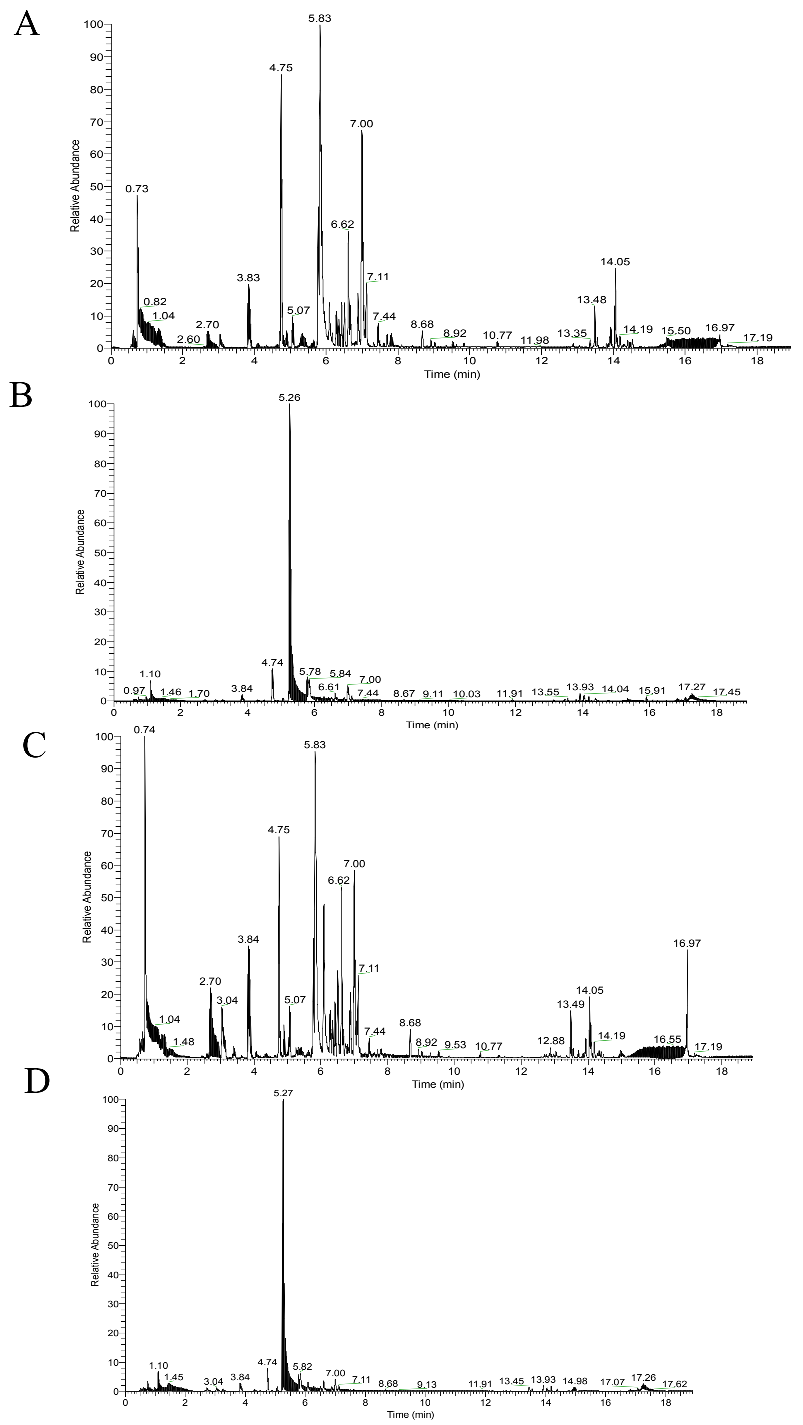

Figure S4. UPLC Base peak chromatogram of the ethanol extract of green tea, A: negative mode; B, positive mode and aqueous extract of green tea, C, negative mode; D, positive mode.

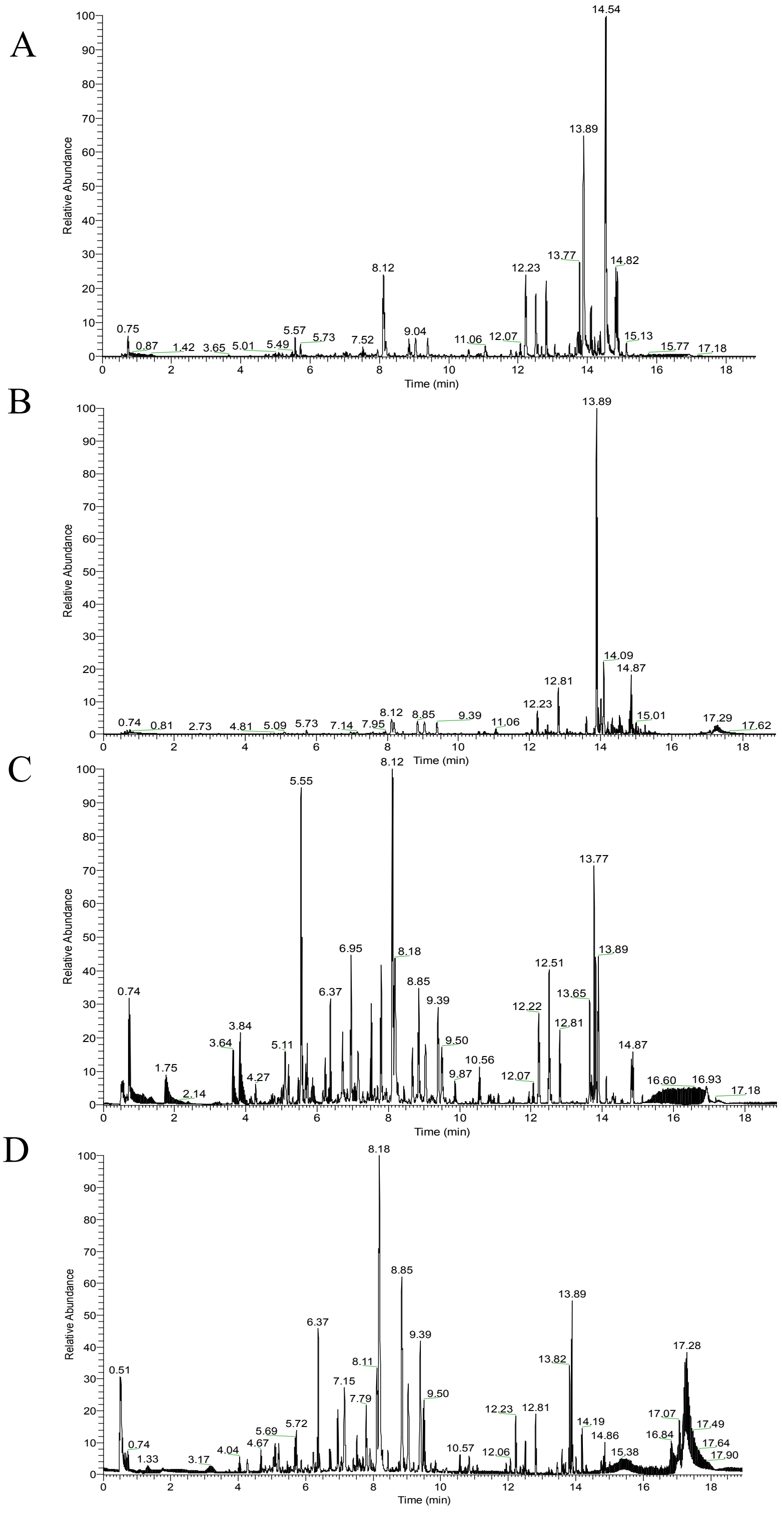

Figure S5. UPLC Base peak chromatogram of the ethanol extract of rosemary, A: negative mode; B, positive mode and aqueous extract of rosemary, C, negative mode; D, positive mode.

Table S1. The identified compounds in the selected herbal extracts based on UPLC–HR–ESI– MS/MS analysis.

RT (min)	M-H	M+H	Formula	Mass Error	Mass fragmentation	Tentative compound name
3.63	609.12622		C30H25O15	3.83	108,152,315,423	Cyanidin 3-(6"-caffeylglucoside)
14.21	373.20172		C22H29O5	3	297,315	11,12-Dimethylrosmanol
6.37	225.1127	227.12737	C12H17O4	2.5	135,179	12-hydroxyjasmonic acid
6.5	163.0390629		C9H7O3	-5.89		2-Hydroxycinnamic acid
12.45		315.08585	C17H15O6	-1.47	135,168	3,7-Dihydroxy-5,6-dimethoxyflavone
11.95	287.2228	289.23676	C16H31O4	4	112,174,248	4,12-Dihydroxy-hexadecanoic acid
9.77		177.05455	C10H9O3	-0.3	81,121	4-Methylumbelliferone
13.1	313.0719	315.0859	C17H13O6	3.85	255,283	5,6-Dihydroxy-3,7-dimethoxyflavone
14.26		329.1015	C18H17O6	-1.3	243,271,299,313	5-Hydroxy-4,6,4'-trimethoxyaurone
13.49	389.197		C22H29O6	2.96	285,303,345	6,7-Dimethoxy-7-epirosmanol
13.82	359.1861	361.2002	C21H27O5	2.22	227,283	7-Methylrosmanol
10.89		301.0702	C16H13O6	-1.5	153,258,286,	8-C-Methylkaempferol
11.51	329.23315		C18H33O5	2.7	112,145,211,	9,10,11-trihydroxy-12-octadecenoic acid
7.82	413.122		C22H21O8	-2.69		Aciculatin
14.17		239.20007	C15H27O2	-0.487	81,91,105,107,184	alpha-Bisabolol oxide C
5.01	451.21881		C27H31O6	16.03	293	Amoradinin
10.57	269.04538	271.05969	C15H11O5	-0.41	119,153,163,204,243	Apigenin
8.69	517.09833	519.11328	C24H23O13	-0.037	271,519	Apigenin 5-(6"-malonylglucoside)
9.27	593.13055		C30H25O13	2.66	161,179,263,269,283	Apigenin 7-(6"-E-Caffeoylglucoside)
8.99	473.10898	475.12299	C23H23O11	-0.49	121,271	Apigenin 7-O-(2"-O-acetylglucoside)
7.88	431.09827	433.11197	C21H21O10	-0.95	271	Apigenin 7-O-glucoside
9.64	559.10876	561.12244	C26H25O14	-1.44	271,517	Apigenin-7-O-(6``-malonyl-4``-acetyl-glucoside)
5.64	401.14542		C18H25O10	2.98	121,161,174,269,327	Benzyl O-[arabinofuranosyl-(1->6)-glucoside]
0.66	215.0321		C12H7O4	-8.2	108, 157	Bergapten
8	373.22165		C19H32O7	3.25		Blumenol C glucoside

5.27		195.0875	C8H11N4O2	0	123,138,163,	Caffeine
4.18	311.041		C13H11O9	3.8	135,179	Caffeoyl tartaric acid
14.54	331.1911	333.2051	C20H27O4	2.24	287	Carnosic acid
14.82	331.1912	333.2054	C20H27O4	2.42	259	Carnosic acid isomer
13.89	329.1753	331.1896	C20H25O4	1.77	201,285	Carnosol
14.31		207.17412	C14H23O	-1.07	93,107,123,135,153,100	Carylophyllene oxide
7	441.0822		C22H17O10	1.26	125,169,289	Catechin gallate
7.24	521.1303		C24H25O13	2.4	161,285,447	Centaurein
5.05	353.08759		C16H17O9	2.49	135,179,191,	Chlorogenic acid
4.27	353.0879	355.1018	C16H17O9	3.34	135,191,307	Chlorogenic acid isomer
5.69	355.10287	357.11722	C16H19O9	1.44	134,149,193,	cis-GMCA
7.04	567.2085		C27H35O13	2.2	112,329	Citrusin B
8.37	371.1351		C17H23O9	3.9	135,161,197,269,311	Citrusin E
6.9	341.12421		C16H21O8	3.2	133,161,174,300,330	Coniferin
6.17	325.0925	327.10675	C15H17O8	2.17	119,163	Coumaroyl glucose
5.2	353.0878	355.1017	C16H17O9	3.09	135,173,179,191	Cryptochlorogenic
12.64		153.12721	C10H17O	-1.1	81,93,107,135	Dihydrocarvone
6.23	371.0981		C16H19O10	2.09	109,135,203,269,313	Dihydroferulic acid 4-O-glucuronide
7.64	515.11938		C25H23O12	1.89	179,191,353,375	Di-O-caffeoylquinic acid
7.44	515.11945		C25H23O12	2.03	161,179,191,300,353	Di-O-Caffeoylquinic acid isomer 1
7.95	515.11938		C25H23O12	1.89	179,191,353,375	Di-O-caffeoylquinic acid isomer 2
12.07	299.0559	301.0703	C16H11O6	3.09	256,284	Diosmetin
5.51		227.12531	C12H19O4	-10.9	91,147,177	epi-4'-hydroxyjasmonic acid
7.8	455.0987		C23H19O10	3.11	125,183,289,	Epicatechin 3-O-(4-O-methylgallate)
4.75	305.06689		C15H13O7	3.79	125,137,167,177,219	Epigallocatechin
5.83	457.0772		C22H17O11	1.405	125,169,179,305	Epigallocatechin Gallate
6.09	457.0772		C22H17O11	1.405	125,169,179,305	Epigallocatechin Gallate isomer
5.57	305.0701		C15H13O7	14.72	180,207,235	Epigallocatechin isomer
11.06	345.1704	347.1848	C20H25O5	2.28	201,271,301	Epirosmanol
10.92	359.0774	361.0915	C18H15O8	3.55	179,258,286,329	Eupatin

12.35		375.107	C19H19O8	-1.1	182,227,317	Eupatoretin
12.61	343.0822	345.0967	C18H15O7	2.94	270,298	Eupatorin
8.42	193.04974	195.06505	C10H9O4	1.061	93,133	Ferulic acid
0.73	533.1724		C26H29O12	13.143	191,341,	Flavonol 3-O-D-xylosylglycoside
5.07	579.1516		C30H27O12	3.19	109,137,245,289	Gambiriin A1
3.84	153.0183		C7H5O4	0.09	108	Gentisic acid
2.7	331.0672		C13H15O10	3.706	169	Glucogallic acid
1.85	132.1026798		C6H14NO2	5.8		Isoleucine
8.1	461.10904		C22H21O11	2.6	209,255,283,298,313	Isorhamnetin 7-rhamnoside
6.88	477.104		C22H21O12	2.5	161,174,191,248,299	isorhamnetin-3-glucoside
12.82	283.0609	285.0753	C16H11O5	2.65	283	Isorosmanol
10.77	285.04047		C15H9O6	3.87		Kaempferol
7.5	447.09375		C21H19O11	3.49	227,255,285	Kaempferol galactoside isomer 2
6.62	593.151		C27H29O15	1.52	301	Kaempferol 3-galactoside-7-rhamnoside
7.44	593.152		C27H29O15	3.17	285	Kaempferol 3-glucosyl-(1->2)-rhamnoside
7.15	461.0728	463.0866	C21H17O12	2.94	285	Kaempferol 3-glucuronide
6.29	563.1409		C26H27O14	2.48	125,169,305,316,353	Kaempferol 3-rhamnosyl-(1->4)-xyloside
6.88	577.1564		C27H29O14	2.05	300	Kaempferol 7,4'-dirhamnoside
7.18	447.0934	449.10718	C21H19O11	2.8	285	Kaempferol galactoside
7.7	447.0938		C21H19O11	3.49	169,227,255,284,305,000	Kaempferol galactoside isomer
7.13	447.0938		C21H17O12	3.49		Kaempferol galactoside isomer 3
0.75	491.1617		C26H19O10	14	179,341,377	Lavandupyrone A
2.01	132.1026798		C6H14NO2	5.8		Leucine
3.83	305.0665		C15H13O7	3.11	125,137,165,219,261	Leucocyanidin
8.24	461.23932		C22H37O10	2.59	145,174,273,301,343	Linalool 3-[rhamnosyl-(1->6)-glucoside]
9.5	285.0404		C15H9O6	3.77	133,180,248	Luteolin
8.18	461.0732	463.0869	C21H17O12	3.7	285	luteolin 3'-O-glucuronide
8.85	503.0831	505.0967	C23H19O13	2.09	255,284,286,399	Luteolin acetylglucuronide
9.39	503.0836		C23H19O13	3.18	146,174,255,284,359	Luteolin acetylglucuronide isomer 1
9.04	503.0831	505.0969	C23H19O13	-1.5	285	Luteolin acetylglucuronide isomer 2

9.45	593.18768	595.2015	C28H33O14	2.34	285	Luteolin p-coumarylglucoside
10.57	329.0668		C17H13O7	3.61	179,271,299	Malvidin
11.84	359.0775		C18H15O8	3.8	202,286,314	Myricetin 3,3',4'-trimethyl ether
6.29	625.14178	627.1554	C27H31O17	0	316	Myricetin 3-rhamnoside-7-glucoside
6.35	479.0833		C21H19O13	2.63	271,316	Myricetin glucoside
6.43	479.08298	481.0973	C21H19O13	2	271,316	Myricetin glucoside isomer
6.96	463.08847	465.1023	C21H19O12	2.95	271, 316	Myricitrin
14.99		223.2054	C15H27O	-1	91,93,123,184	Nerolidol
5.11	325.0926		C15H17O8	2.35	121,163	O-Coumaric acid 2-glucoside
3.74	220.1178573		C9H18NO5	-0.4		Panthothenic Acid
5.87	337.093		C16H17O8	3.45	93,119,163,173	p-Coumaroylquinic acid
11.29	166.0834742		C9H12NO2	-16.74		Phenylalanine
11.3		135.11754	C10H15	5.2		p-Mentha-1,3,8-triene
4.3		207.1032	C12H15O3	1.62	91,115,118,130,132,	p-Methoxycinnamic acid ethyl ester
4.9	577.1358		C30H25O12	3.02	119,163,191,305,337	Procyanidin B2
0.72	116.0714258		C5H10NO2	7.07		Proline
12.77	373.09299	375.10663	C19H19O8	-0.814	151,261,271,299,317,345,	Quercetagetin 3,7,3',4'-tetramethyl ether
7.22	493.09879		C22H21O13	2.27	181,271,287,315,	Quercetagetin 7-methyl ether 6-glucoside
3.05	609.12616		C30H25O14	3.74	125,177,305,329,423	Quercetin 3-(3-p-coumaroylglucoside)
9.28	591.18555	593.1856	C28H33O14	-1.5	285	Quercetin 3,3'-dimethyl ether 7-rutinoside
13.2		359.1119	C19H19O7	-1.8	162,270,298,343,381	Quercetin 3,5,7,3'-tetramethyl ether
7.48	433.07755	435.0916	C20H17O11	2.33	255,271,301	Quercetin 3-arabinoside
7.02	463.0882	465.1021	C21H19O12	2.69	301	Quercetin 3-galactoside
6.5	595.13074	597.1444	C26H27O16	0	301	Quercetin 3-glucosyl-(1->2)-xyloside
7.11	463.0891		C21H19O12	4.33	285,300,330	Quercetin 3-O-glucoside
6.47	463.08835		C21H19O12	2.69	134,173,301,367,389,	Quercetin 3-O-glucoside isomer
6.88	609.14685	611.16	C27H29O16	0	301,316,449	Quercetin 3-rhamnoside-7-glucoside
0.74	191.0552		C7H11O6	0.2		Quinic acid
6.72	541.1346	543.1486	C27H25O12	0.97	135,179,197,211,255	Resveratrol 4'-(6-galloylglucoside)
13.65	343.1549		C20H23O5	2.56	216,243,299	Rosmadial

12.51	345.1704	347.1845	C20H25O5	2.11	168,283	Rosmanol
12.23	345.1702	347.1844	C20H25O5	1.5	283,301	Rosmanol isomer
14.31	315.1964		C20H27O3	2.97	201,285	Rosmaridiphenol
8.12	359.077	361.09116	C18H15O8	2.3	135,161,197	Rosmarinic acid
4.54	299.11365		C14H19O7	3.1	261,293	Salidroside
3.65	417.0803		C20H17O10	-3.1	99,135,179,219	Salvianolic acid G
7.59	577.15741		C27H29O14	3.86	169,272,	Scutellarein 7,4'-dirhamnoside
4.78	323.07724		C15H15O8	2.75	112,153,161,174	Skimmin
9.5	285.0406	287.05484	C15H9O6	4.1	133	Theogallin
3.05	343.0671		C14H15O10	3.22	191	Tomenin
5.44	383.09628		C17H19O10	-2.59	151,167,229,305	Tonghaosu
6.74	355.103	357.11722	C16H21O9	-0.789	121,177,234	trans-GMCA
4.27	205.095701		C11H13N2O2	-7.08		Tryptophan
5.73	387.1658		C18H27O9	2.1	387	Tuberonic acid glucoside
3.76		163.05795	C6H11O5	-13.18	107,117,121,122,132	Umbelliferone
6.18	461.1669		C20H29O12	3.35	131,174,248,301	Verbasoside

Table S2. The distinctive metabolites with significant accumulation in Rosemary

Name	p value	log_2 fold change
11,12-Dimethylrosmanol	1.93E-09	10.961
6,7-Dimethoxy-7-epirosmanol	0.00049338	11.228
7-Methylrosmanol	0.0039643	7.9667
Quercetin 3,3'-dimethyl ether 7-rutinoside	0.0010523	3.37
Carnosic acid	1.88E-05	10.18
Carnosol	0.00070046	10.32
Luteolin p-coumarylglucoside	0.00090372	2.2635
Diosmetin	0.00085396	10.302
Epirosmanol	0.0042544	6.336
Isorosmanol	0.0031406	7.3951
luteolin 3'-O-glucuronide	2.28E-05	7.8784
Luteolin acetylglucuronide	7.41E-05	8.5762
p-Coumaroylquinic acid	0.0018996	4.8551
Rosmaridiphenol	1.19E-07	4.7265
Rosmadial	0.0050944	8.8868
Rosmanol	1.31E-05	9.4685
Rosmarinic acid	0.0031302	4.2044
